# A Model of Pressure Distribution Under Peripherally Secured Foam Dressings on a Convex Surface: Does This Contribute to Skin Graft Loss?

**Published:** 2010-02-23

**Authors:** Lara Wetton, Johnny Kwei, John Kippen, Sean Nicklin, Mark Gillies, William R. Walsh

**Affiliations:** Research Laboritories, University of New South Wales, and The Prince of Wales Hospital, Australia

## Abstract

**Background:** Successful skin grafting requires multiple factors for success. An even distribution of constant pressure exerted upon the graft is necessary for successful graft take. It is well known that excessive pressure on a graft causes ischemia and may result in the failure of graft take. The aim of this study was to demonstrate the variation in skin pressure (tension) on curved surfaces, particularly relating to apical pressure on such surfaces at standard atmospheric pressure. **Methods:** A synthetic Sawbone skull model was used to determine skin tension over a curved surface. A 10-cm diameter circle was centered on the parietal eminence, the area of maximum curvature. Peripheral screws gave fixed reproducible points to secure the foam dressing. Open-cell VAC dressing foam was used and calibrated Tekscan sensors were used to determine pressure variation under the foam dressing. **Results:** Five hundred pressure readings were obtained for the unscored foam, and an additional 500 for the cross-scored foam. In the unscored foam, the pressure under the dressing was significantly higher at the apex. Cross-scoring the foam reduced the pressure, with the greatest reduction being at the apex. The pressure under the foam dressing was maximal at the apical point (95% confidence interval). **Conclusion:** Higher contact force at the apex of a curved graft bed may explain skin graft loss. Unequal pressure distribution can be reduced and equalized by scoring the foam.

Although the mechanism of successful skin grafting is incompletely understood, one of the basic requirements is the application of a constant and even distribution of pressure on the graft.^1^ Weiner and Moberg^2^ suggested that the ideal pressure is between 16 and 25 mm Hg. Appropriate pressure ensures proper contact of the graft to the bed, reduces shearing forces, and decreases the likelihood of seroma and hematoma formation under the graft. However, excessive pressure has been suggested to cause the graft to break down,^3^ perhaps by prevention of revascularization, through decreased plasma imbibition. Several methods, including tie-over bolsters and foam dressing stapled into place, are available for dressing skin grafts.^4^

Foam dressing for applying an even pressure distribution on a skin graft was described as early as the 1920s. Some authors have proposed that foam bolsters can provide a uniform distribution of pressure, even over curved surfaces.^2^ Skin grafting on a curved surface such as the skull is a long-standing technique and was even practiced by the ancient Egyptians.^5^ More recently, it has been reported that foam dressings peripherally secured with staples only, do not apply an even pressure distribution over the entire graft, but a ring-shaped pressure distribution with minimal projection, with least pressure applied over the central portion in a circular dressing over a flat surface.^6^

The pressure variation applied by foam dressings over a curved surface is, to the author's knowledge, as yet unknown. It has been observed that central necrosis of skin grafts is a complication over curved surfaces such as the skull.^7^

It is postulated that the observed necrosis in the center of the skin graft is due to the increased pressure of the foam over the graft at the apex or where the highest point of curvature should occur, resulting in central pressure necrosis.

The goal of this study was 2-fold; first, to establish whether the pressure is highest at the apex of a foam dressing that is peripherally secured (because this is a common method of dressing a graft), and second, if the pressure should be highest at the apex and whether this can be decreased and equalized by cross-scoring the foam.

## MATERIALS AND METHODS

In this study, we used VAC dressing foam as the dressing over the parietal eminence of a synthetic Sawbone skull. The parietal eminence of the skull has the area of maximum curvature. VAC foam was used; however, the pressure readings took place at standard atmospheric pressure of 760 mm Hg.

A 10-cm diameter was centered over the parietal eminence and fixed with peripheral screws, which gave fixed reproducible points to secure subsequent synthetic grafts. The synthetic graft was unfenestrated so as to mimic a full-thickness skin graft.

**Figure 1:**
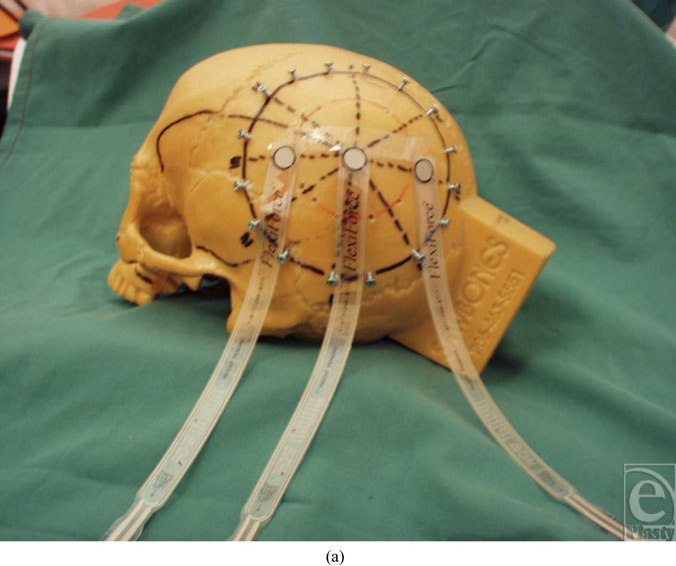

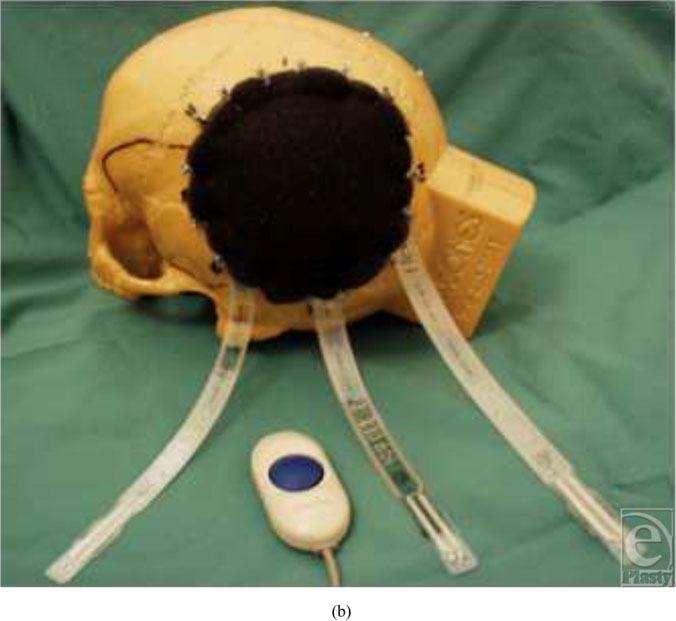
(a) Photograph showing some of the areas where pressure sensors were applied. (b) VAC dressing fixed into place.

Pressure between the synthetic graft and the synthetic skull was detected with calibrated sensors (Tekscan, Boston, MA). These were placed at 5 locations, namely, anterior, posterior, superior, inferior, and at the apex (Fig 1). Subsequent pressure readings were generated from these areas.

One hundred static contact force measurements were made at each point. The foam was then scored and 100 comparison contact force measurements were made. The number of readings was well within statistical significance. (The sequence was then repeated 5 times.) After moving the sensors to the superior, posterior, anterior, and inferior positions and at the apex, the 5 runs were repeated. This generated 500 readings for each point. Pressure readings were measured in kilopascals (kPa) and the load across the graft in newtons (N).

## RESULTS

**Figure 2:**
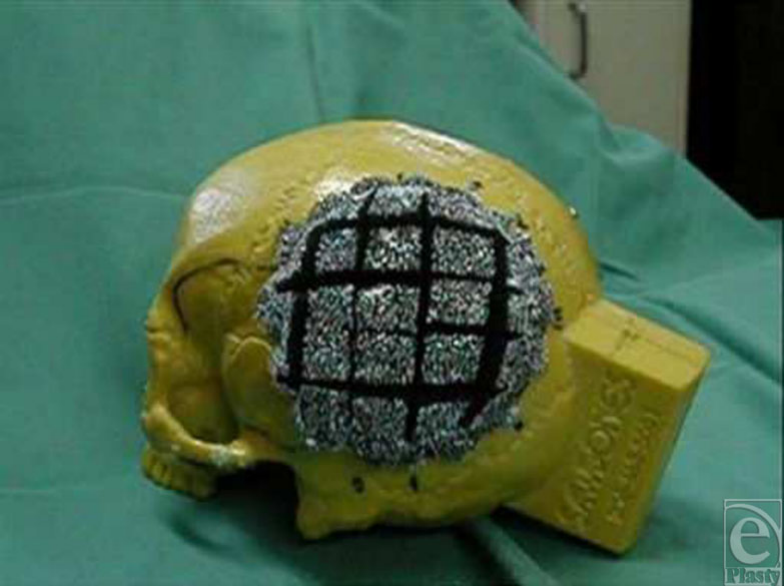
Cross-scored foam.

Five hundred pressure readings in total were produced for the unscored foam and 500 readings for the cross-scored foam (Fig 2). A paired Student *t* test was performed for statistical comparison. The contact forces generated were consistent within experiments.

**Figure 3:**
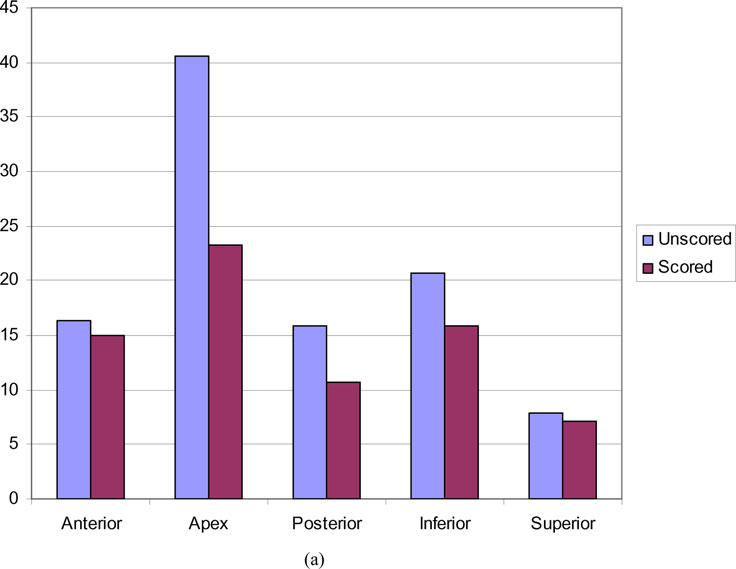

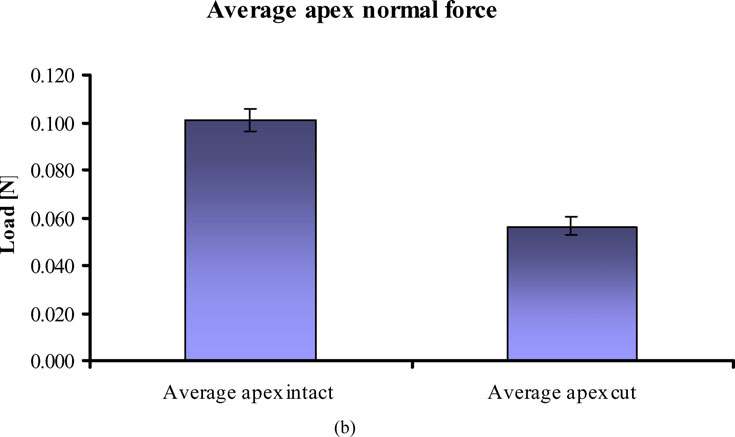
(a) Graphic representation of the pressures recorded (kPa) at 5 different points in scored and unscored foam dressings. (b) Graph showing standard deviation for a single run (*P* < .05).

In unscored foam, pressure was not equal across the foam dressing, with statistically significant higher contact forces recorded at the apex. Cross-scoring of the foam resulted in a reduction in all measured contact forces and more equal distribution of pressure over the curved surface. Apical reduction in pressure was greatest, whereas the least such reduction was demonstrated at the anterior points (Table 1 and Fig 3).

## DISCUSSION

The purpose of this study was to demonstrate a variation in skin pressure distribution across a curved graft surface at standard atmospheric pressure. This was demonstrated effectively when multiple readings were obtained from a surface with maximal curvature at an apical point. Factors for the production of such skin pressure could include stress loads on end points, vascular compromise at apices, and the consequence of soft tissue under tension.

Skin is viscoelastic and as a result of mechanical response to loading involves both a viscous component associated with energy dissipation and an elastic component with energy storage. The mechanical properties of skin are related to the structure and properties of collagen, elastic fibres, and proteoglycans. Thus, the way skin behaves is determined by the degree of stretch. There are 3 phases: elastic fibres are active in strains of 0.3, collagen fibrils dominate in strains of 0.3 to 0.6, and finally the elastic component dominates, and may stretch the cross-linked collagen.^8^

In this laboratory study, we inadvertently address skin stretch because it can be deduced that skin stretch would be greatest at the apex of a curved surface. Interestingly, there are alterations in skin microstructure and microcirculation when skin is stretched. For example, collagen fibres in the dermis realign in the direction of the stretching force such that they are perpendicular to the wound margin.^9^,^10^ Whether this significantly weakens the integrity of the dermis in this setting is yet unknown. However, in other settings, it has been suggested that skin stretching leads to decreased dermal cell density, which could lead to impaired quality of skin and reduced biomechanical strength.^11^

It has been demonstrated that skin stretching results in a decrease in laser Doppler flowmetry signals and transcutaneous oximetry values (ie, both decreases blood flow and oxygen availability).^9^ If the stretch is released, the laser Doppler flowmetry values quickly return to near-normal values in nonundermined skin; however, if the skin is undermined, there is a permanent vascular compromise sustained. Undermined skin that is stretched is more likely to undergo skin necrosis that nonundermined stretched skin.^9^ This is applicable to our model because it suggests that undermined skin on a curved surface (such as the skull) would have poor outcomes.

Scoring the foam significantly reduced the pressure at the apex, which may potentially arrive from the redistribution of tension and the redistribution of pressure such that the microvascular blood supply to the skin is not impaired. This may similarly be the case if the skin itself was fenestrated.

This study was performed with the use of simple foam dressings at standard atmospheric pressure. It would be worth repeating this experiment with a VAC dressing when subatmospheric pressure is applied. The negative pressure that would be applied (125 mm Hg) may alter the pressure readings obtained from the 5 locations measured on the Sawbone skull. Furthermore, different levels of subatmospheric pressure could be used, which may have a significant effect on the measured pressure gradients.

In this case, the synthetic graft was not fenestrated because the aim was to replicate a full-thickness graft. However, a fenestrated synthetic graft, which would replicate a split-skin graft, may also be used and may impact the distribution of pressure to the underlying wound bed.

While these results are demonstrated in the Sawbone model, we cannot extrapolate to the in vivo setting. There may be other important confounding variables including altered vascular supply, dynamic forces, and securing devices such as sutures or staples. Perhaps, once the exact degree of pressure for optimal graft take on a curved surface is established, it could then be replicated on patients who have had a skin graft. It would then be possible to tailor the dressing to an ideal pressure distribution and routinely achieve 100% graft take.

## CONCLUSION

This laboratory model demonstrated that there is an unequal pressure distribution exerted on a skin graft covering a curved surface when the frequently used foam dressing is used. (This is clinically significant because full-thickness loss of skin from the scalp is a common occurrence in cancer, burn, and trauma patients.^12^,^13^) Higher contact force at the apex may explain skin graft loss. Unequal pressure distribution can be reduced and equalized by scoring the foam, because scoring reduces the entire pressure, with the pressure at the apex having the greatest reduction. We are planning a follow-up study in a cadaver model with time-based pressure changes on the scalp, which may more closely reflect a clinical setting.

## Figures and Tables

**Table 1:** Mean pressure (kPa) readings across scored and unscored substitute graft (*N* = 500) at atmospheric pressure of 760 mm Hg

	Anterior	Apex	Posterior	Inferior	Superior
Unscored	16.3655	40.5153	15.8886	20.6235	7.8816
Scored	15.0397	23.2825	10.718	15.8025	7.1291
% Reduction	8.10*	42.53*	32.54*	23.37†	9.55†

*P≤ .001.

†*P* = .001
